# Whole‐exome sequencing analysis of juvenile papillomatosis and coexisting breast carcinoma

**DOI:** 10.1002/cjp2.190

**Published:** 2020-12-02

**Authors:** Timothy M D'Alfonso, Fresia Pareja, Arnaud Da Cruz Paula, Mahsa Vahdatinia, Andrea Gazzo, Lorenzo Ferrando, Edaise M da Silva, Esther Cheng, Lisa Sclafani, Sarat Chandarlapaty, Hong Zhang, Syed A Hoda, Hannah Y Wen, Edi Brogi, Britta Weigelt, Jorge S Reis‐Filho

**Affiliations:** ^1^ Department of Pathology Memorial Sloan Kettering Cancer Center New York NY USA; ^2^ Department of Surgery Memorial Sloan Kettering Cancer Center New York NY USA; ^3^ Department of Internal Medicine University of Genoa Genoa Italy; ^4^ Department of Pathology and Laboratory Medicine Weill Cornell Medicine New York NY USA; ^5^ Department of Medicine Memorial Sloan Kettering Cancer Center New York NY USA

**Keywords:** juvenile papillomatosis, *PIK3CA*, breast carcinoma

## Abstract

Juvenile papillomatosis (JP) of the breast is a rare benign mass‐forming lesion occurring in young women, which is histologically characterized by a constellation of proliferative changes and large cysts, giving it the gross appearance of Swiss cheese. A subset of patients with JP report a family history of breast carcinoma and/or coexisting or subsequent breast carcinoma. We performed whole‐exome sequencing of the hyperplastic epithelial component of three JPs, including one with coexisting ductal carcinoma *in situ* (DCIS) and invasive ductal carcinoma of no special type (IDC‐NST). JPs harbored clonal somatic *PIK3CA* hotspot mutations in two cases. In the JP with coexisting DCIS and IDC‐NST, these lesions were clonally related to the associated JP, sharing a clonal *PIK3CA* E542K somatic hotspot mutation. JP showed a paucity of copy number alterations, whereas the associated DCIS and IDC‐NST showed concurrent 1q gains/16q losses, hallmarks of estrogen receptor (ER)‐positive breast cancers. We observed JP to harbor a dominant aging‐related mutational signature, whereas coexisting DCIS and IDC‐NST showed greater exposure to an APOBEC signature. Taken together, our findings suggest that, at least in a subset of cases, JP might constitute the substrate from which DCIS and invasive breast carcinomas develop.

## Introduction

Juvenile papillomatosis of the breast (JP) is a rare benign mass‐forming lesion usually occurring in women in their 20s to 30s that often mimics fibroadenoma clinically [1, 2]. Histologically, JP is characterized by a constellation of proliferative and nonproliferative changes, the most distinct being abundant large cysts, giving the gross appearance of Swiss cheese [1]. Since the description of JP as a distinct entity by Rosen [1], multiple studies have reported a significant family history of breast carcinoma cancer in patients developing JP [2, 3, 4], suggesting a relationship between JP and breast carcinoma. Furthermore, patients with JP have coexisting breast carcinoma or subsequently develop carcinoma in approximately 10% of cases [4, 5]. Recently, Guillet *et al* reported recurrent *PIK3CA* or *AKT1* hotspot mutations in benign JP lesions in the first published study of the molecular features of JP, expanding the spectrum of benign breast lesions with these mutations [3]. Nonetheless, the genetic relationship between JP and coexisting breast carcinoma has not yet been documented. Herein, we sought to characterize the repertoire of genetic alterations of JP by whole‐exome sequencing (WES) and to study the clonal relationship between JP, ductal carcinoma *in situ* (DCIS), and invasive carcinoma in one case where these components coexisted.

## Materials and methods

### Subjects and samples

Following approval from the Institutional Review Boards (IRB), formalin‐fixed paraffin‐embedded tissue blocks were retrieved from the pathology archives of the authors' institutions. Patient consents were obtained if required by the IRB protocols of the different institutions. Samples were anonymized prior to tissue processing. Our cohort included three JP lesions (JuP1, JuP2, and JuP3), including one case (JuP3) with coexisting invasive ductal carcinoma of no special type (IDC‐NST) and DCIS. Slides were reviewed by three pathologists (TMD, FP, and MV). The hyperplastic epithelial components of JP, and DCIS and IDC‐NST components of case JuP3 were separately microdissected from 10 to 15 8‐μm thick histological sections under a stereomicroscope (Olympus SZ61, Tokyo, Japan), as previously described [6]. DNA was extracted from lesion/tumor and matched benign non‐hyperplastic breast parenchyma away from the JP using the DNAeasy Blood and Tissue Kit (Qiagen, Hilden, Germany), following manufacturers' instructions. DNA samples were subjected to WES at the Integrated Genomics Operation at Memorial Sloan Kettering Cancer Center and analyzed.

### Whole‐exome sequencing analysis

Reads were aligned to the reference human genome GRCh37 using the Burrows‐Wheeler Aligner (BWA v0.7.15) [7]. The Genome Analysis Toolkit (GATK. V3.1.1) [8] was employed for local realignment, duplicate removal and base quality recalibration. Somatic single nucleotide variants (SNVs) were detected with MuTect (v1.0) [9], indels with Strelka (v2.0.15) [10], Varscan2 (v2.3.7) [11], Scalpel (v0.5.3) [12], and Lancet (v1.0.0) [13] as previously described [6]. SNVs and indels outside of the target regions were filtered out, as were SNVs and indels for which the variant allele fraction (VAF) in the tumor sample was <5 times that of the paired normal VAF, and SNVs and indels found at >5% global minor allele frequency of dbSNP (build 137), as previously described [14]. Only somatic mutations with a depth ≥20 reads in the respective normal samples were considered [14]. All mutations were manually inspected using the Integrative Genomics Viewer (IGV) [15]. The cancer cell fraction (CCF) of each mutation was inferred using ABSOLUTE (v1.0.6) [16], as previously described [17, 18]. Mutations were cataloged as clonal if their probability of being clonal was >50% [19], or if the lower bound of the 95% CI of its CCF was >90% [20]. Copy number alterations and loss of heterozygosity were determined using FACETS [21]. Mutations targeting hotspot loci [22] were annotated as previously described [20]. Mutational signatures were inferred using SigMA [23] based on all synonymous and nonsynonymous somatic mutations, as previously reported [24]. A mutation‐based phylogenetic tree of the JP and associated DCIS and IDC‐NST of case JuP3 was constructed using Treeomics [25], based on all synonymous and nonsynonymous mutations identified. Mutations were considered shared when present in different histologic components, and private when present in only one of the components. Clonal relatedness between the JP and coexisting DCIS and IDC‐NST of case JuP3 were assessed using ‘clonality index’ (CI), which is the probability of the mutations shared between different lesions not co‐occurring by chance [26, 27]. We used a previously established method [26, 27], where CI is defined as follows: $\mathrm{CI}=-\log_{10}\prod_{m=1}^{M}P{\left(X\right)}_{m}.$ The probability of identifying a given mutation in two samples is defined by the binomial probability *P*(*X*) = *C* ^*k*^_*n*_ *p* ^*k*^(1 − *p*)^*n*−k^, *n* = 2, *k* = 2, where *p* represents the frequency of a given mutation and *n* represents the number of shared mutations in different components or the average number of mutations identified in the samples. Hence, the probability of identifying a certain set of *M* identical mutations in the different samples is given by $\prod_{m=1}^{M}P{\left(X\right)}_{m}$.

## Results

Case JuP1 was a 43‐year‐old woman without a history of breast carcinoma who presented with a palpable breast mass. She underwent lumpectomy that showed JP. Case JuP2 was a 31‐year‐old woman with a family history of breast carcinoma who was found to have clustered calcifications on screening mammogram. Following biopsies showing fibrocystic changes, she underwent lumpectomy showing JP. JuP3 was a 34‐year‐old woman with a family history of breast carcinoma and a history of fibrocystic changes. Ultrasound revealed an irregular hypoechoic mass that was biopsied revealing IDC‐NST and DCIS. She underwent lumpectomy showing JP in association with the carcinoma.

**Figure 1:**
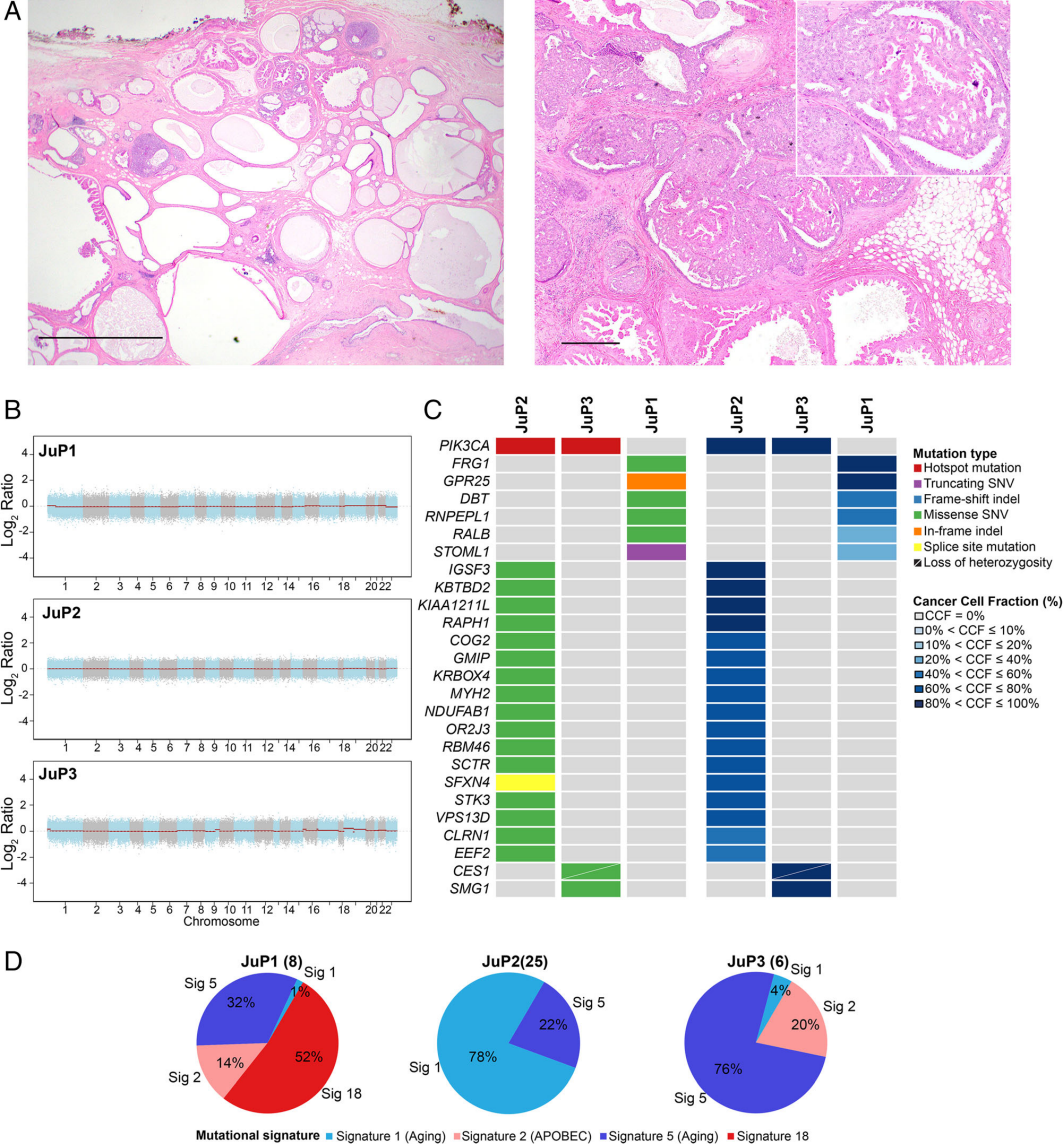
Histologic features and repertoire of genetic alterations of JP. (A) Representative micrographs of H&E stained sections of JP illustrating the characteristic cystic cut surface (left) and florid hyperplasia with papillary and apocrine features with calcifications (right). Scale bars: left, 2 mm; right, 500 μm. (B) Genome plots depicting Log_2_ ratios (*y*‐axis) plotted according to their genomic positions (*x*‐axis). (C) Heatmaps depicting the nonsynonymous somatic mutations identified by whole‐exome sequencing (left) and cancer cell fraction (CCF) (right). Cases are shown in columns and genes in rows. Mutations and CCF are color‐coded according to the legend. (D) Pie charts showing the mutational signatures as inferred by SigMA. The numbers of synonymous and nonsynonymous mutations of each JP lesion are shown in parentheses.

**Figure 2:**
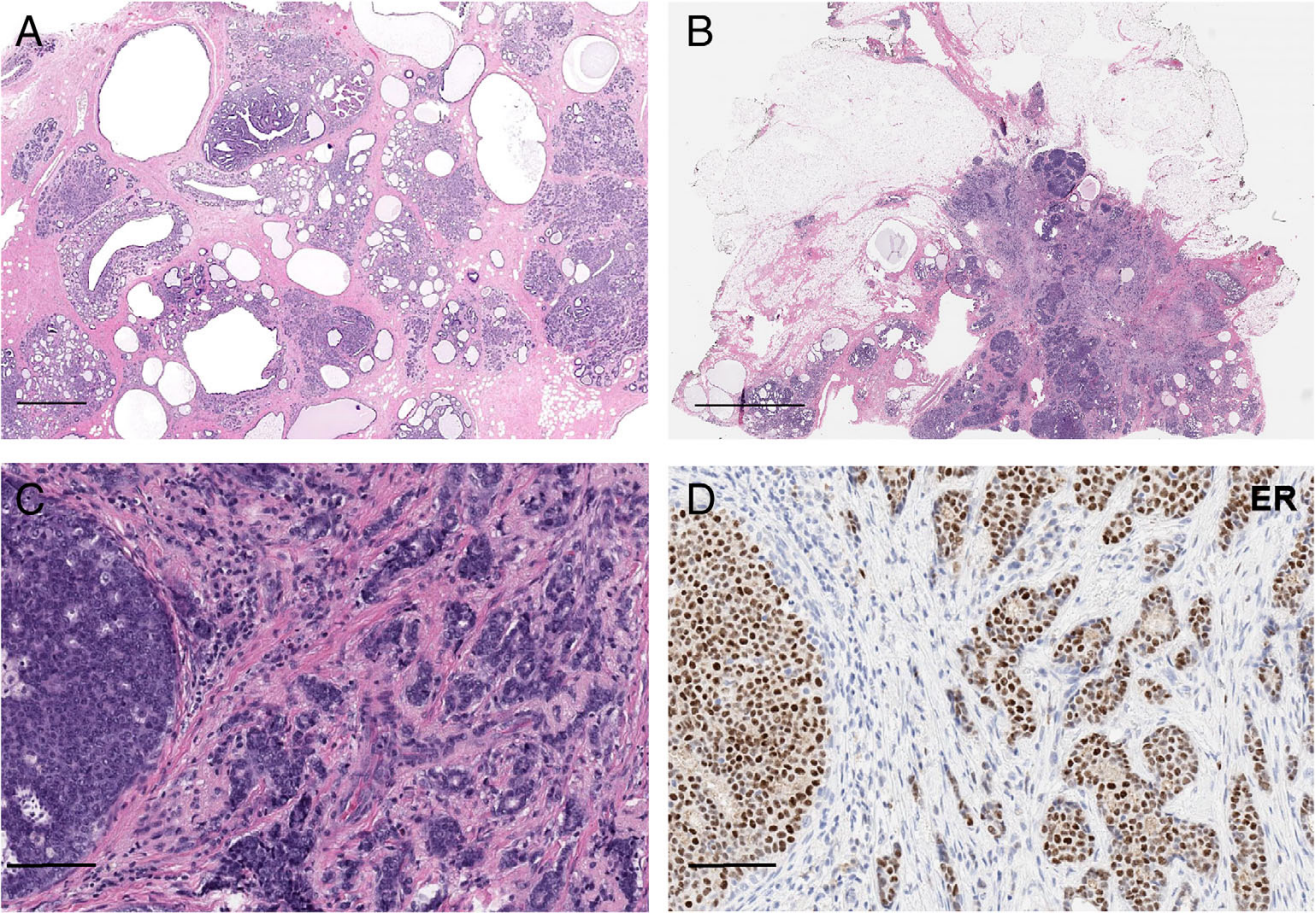
Histologic images of JP and synchronously identified invasive breast carcinoma and DCIS. (A) Representative micrographs of H&E stained sections of JP showing the cystic cut surface and (B) synchronously identified DCIS and IDC‐NST of case JuP3. (C) Higher power magnification shows DCIS adjacent to the IDC‐NST, showing a moderately differentiated IDC‐NST and intermediate‐grade DCIS. (D) Representative micrograph of the ER immunohistochemical analysis showing strong positive staining in IDC‐NST and DCIS. Scale bars: A: 1 mm; B: 3 mm; C, D: 100 μm.

All cases showed classic histologic features of JP including abundant large cysts, ducts with lipid‐laden histiocytes, florid usual ductal hyperplasia, and apocrine metaplasia/hyperplasia (Figure 1A,B). Stromal sclerosis, papilloma/papillary hyperplasia, adenosis, and microcalcifications were present in varying degrees among cases. JuP3 showed a 1.5 cm estrogen receptor (ER)‐positive/HER2‐negative moderately‐differentiated IDC‐NST and DCIS with intermediate to high nuclear grade and necrosis that was intimately associated with benign JP components (Figure 2A–D).

To determine the repertoire of genetic alterations underpinning JP we subjected these three cases to WES. Our analysis revealed that JP shows a paucity of copy number alterations (CNAs) and somatic mutations (Figure 1B,C). Benign JP showed a median of 9 (range, 6–25) total and 6 (range, 3–18) nonsynonymous mutations. We identified somatic clonal *PIK3CA* hotspot mutations in cases JuP2 (H1047R) and JuP3 (E542K). Interestingly, JuP1 was found to harbor a germline *PIK3CA* I391M variant, whose significance is uncertain. No other genes were found to be recurrently mutated. We inferred the mutational signatures using SigMA [23] using all synonymous and nonsynonymous somatic mutations. We observed that mutational signatures ascribed to aging (signatures 1 and 5) were dominant in JuP2 and JuP3 (Figure 1D). In JuP1, signature 5 (aging) was the second most dominant following signature 18, of cryptic origin (Figure 1D).

**Figure 3:**
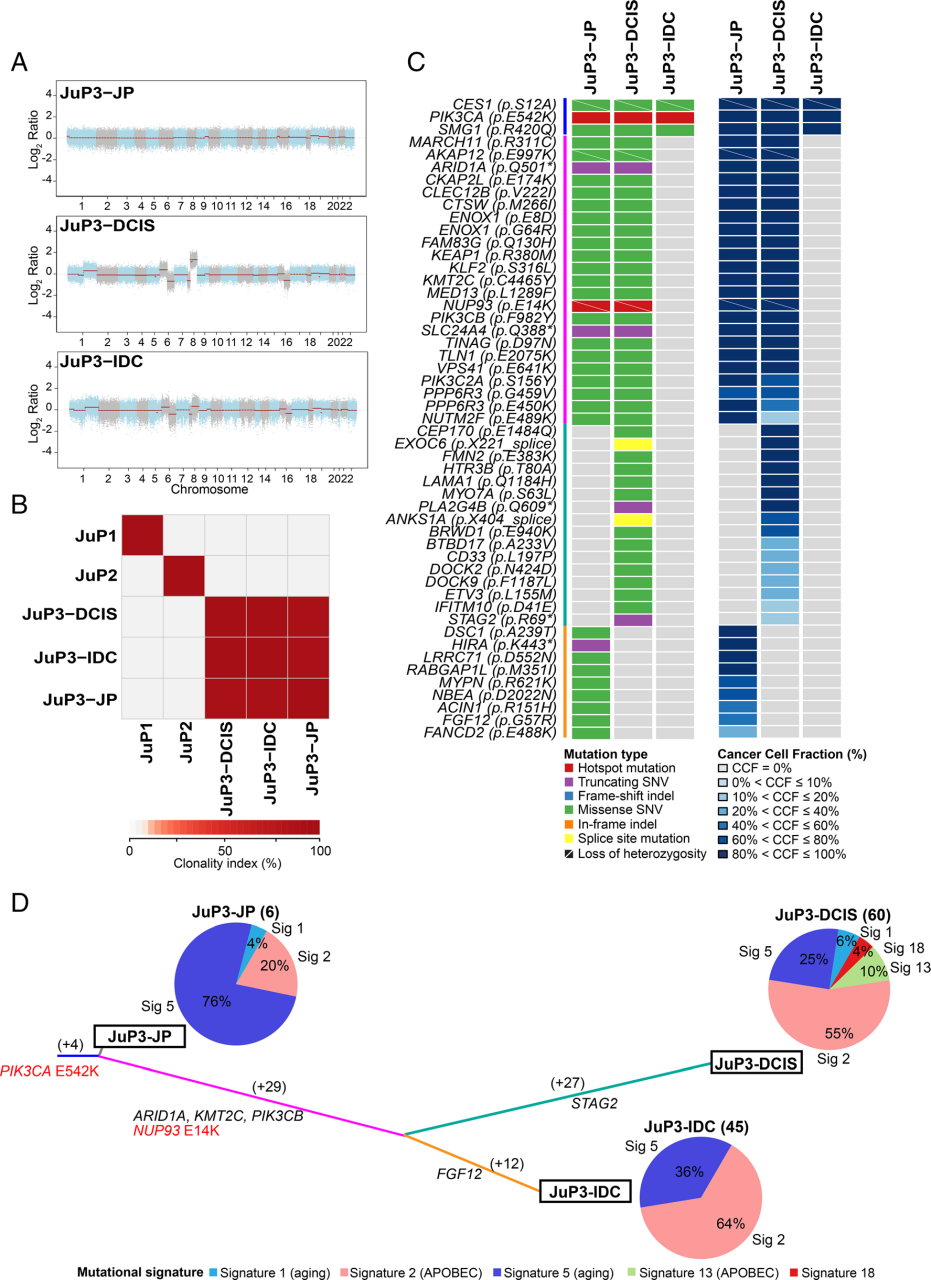
JP and coexisting breast cancer are clonally related. (A) Genome plots depicting Log_2_ ratios (*y*‐axis) plotted according to their genomic positions (*x*‐axis). (B) Pairwise comparison of the clonality index (CI) based on somatic mutations identified in JP lesions JuP1, JuP2 and JuP3 and ductal carcinoma *in situ* (JuP3‐DCIS) and invasive ductal carcinoma (JuP3‐IDC) coexisting with JuP3 identified by whole exome sequencing. (C) Heatmaps depicting the nonsynonymous somatic mutations identified in the different histologic components of case JuP3 (left) and their cancer cell fraction (CCF) (right). Cases are shown in rows and genes in columns. Mutations and CCF are color‐coded according to the legend. (D) Mutation‐based phylogenetic tree of case JuP3 depicting the clonal evolution of JP and synchronously identified DCIS and IDC‐NST. The lengths of the trunk and branches are proportional to the number of synonymous and nonsynonymous mutations shared or private to a given histologic component. Mutational signatures identified in the different histologic components of case JuP3 using SigMA are depicted in pie charts. The numbers of synonymous and nonsynonymous mutations of each histologic component are shown in parentheses.

As a hypothesis generating aim, we sought to determine whether JP could constitute a substrate for the development of breast carcinoma and conducted WES analysis of the separately microdissected JP and coexisting DCIS and IDC‐NST of JuP3. Our analyses revealed that, although the JP component of JuP3 displayed a paucity of CNAs, the synchronously identified DCIS and IDC‐NST showed concurrent 1q gains/16q losses, the hallmark genetic alteration of ER‐positive breast cancer [28] (Figure 3A). We observed that the DCIS and IDC‐NST of JuP3 were clonally related to adjacent JP and shared a clonal *PIK3CA* E542K hotspot mutation and *CES1* (S12A) and *SMG1* (R420Q) missense mutations (Figure 3B,C). DCIS and IDC‐NST shared 29 synonymous and nonsynonymous mutations not seen in JP, including a truncating mutation in the chromatin remodeling gene *ARID1A*, missense mutations in *KMT2C* and *PIK3CB*, and an E14K hotspot mutation affecting *NUP93*, a nucleoporin implicated in cell migration [29] (Figure 3B,C). A subset of mutations found to be subclonal in the DCIS became dominant in the IDC‐NST, including a *PIK3C2A* missense mutation, suggesting that a minor subclone of the DCIS became the dominant clone in the progression from DCIS to invasive breast cancer (Figure 3B,C). The DCIS and IDC‐NST subsequently acquired somatic mutations, with the acquisition of a truncating mutation in the tumor suppressor *STAG2* in DCIS and an *FGF12* missense mutation in IDC‐NST (Figure 3B,C).

Mutational signature analysis revealed that JP harbored a dominant aging‐related signature 5 (signature exposure of 76%) followed by signature 2 (APOBEC; signature exposure of 20%). The DCIS and IDC‐NST from JuP3, however, displayed a greater number of mutations than benign JP as well as a greater mutational signature 2 exposure (DCIS: 55%; IDC‐NST: 64%), suggesting that the development of carcinoma in this case might be associated with an increase in APOBEC mutagenesis (Figure 3D).

## Discussion

Here we provide evidence that JP shows recurrent *PIK3CA* mutations, and that it may constitute the substrate from which DCIS and invasive breast cancers develop, given that we documented clonal relatedness between JP and associated carcinoma.

Mutations in the PI3K‐AKT pathway, which are commonly seen in invasive breast carcinoma, have also been identified in a variety of benign proliferative epithelial lesions, including papillary neoplasms, usual ductal hyperplasia, and columnar cell change, among others [30, 31]. WES analysis of the proliferative components of JP resulted in the identification of clonal *PIK3CA* hotspot mutations in 2 of 3 cases studied. We also observed a germline *PIK3CA* variant affecting the C2 domain in the third case. Although mutations affecting the C2 domain of *PIK3CA* have been reported to increase its kinase activity [32], the I391M germline mutation has a frequency of 6% in the normal population. Hence, its significance is uncertain. Our study confirms the findings of Guillet *et al* [3], who recently reported *PIK3CA* and *AKT1* mutations in 5 of 10 and 2 of 10 cases of JP, respectively. In our study and that of Guillet *et al* [3], it was the epithelial hyperplastic component of JP that was sequenced. It is unclear whether these mutations would be evident in other components of JP. Nonetheless, these studies expand the spectrum of benign breast lesions harboring mutations affecting PI3K‐AKT pathway‐related genes to include JP.

Interestingly, there are rare reports of JP occurring in patients with Cowden syndrome and Proteus syndrome [5], two syndromes affecting genes in the PI3K pathway. Patients with Cowden syndrome, caused by a germline *PTEN* mutation, develop hamartomatous lesions and are at risk of multiple cancers [33]. Proteus syndrome, characterized by progressive asymmetric growth of multiple tissue types, is caused by somatic mosaicism for an activating *AKT1* mutation [34]. The presence of PI3K pathway mutations among these disorders suggests a possible link to the development of JP. Study of such lesions, however, is challenging due to their rarity.

Our study not only confirms the presence of *PIK3CA* hotspot mutations in JP, but also expands our understanding of the molecular relationship between JP and breast cancer, given that here we demonstrated in one case the presence of a clonal E542K *PIK3CA* hotspot mutation shared by separate components of JP, DCIS, and IDC‐NST. The IDC‐NST component was ER‐positive/HER2‐negative with no distinctive histologic features and displayed CNAs typical of ER‐positive disease. Other types of breast carcinoma including invasive lobular carcinoma [5] and secretory carcinoma [1, 35] have been reported in association with JP. Classic lobular carcinomas are usually ER‐positive/HER2‐negative tumors driven by *CDH1* alterations and *PIK3CA* mutations [36], whereas secretory carcinoma is a low‐grade triple‐negative breast cancer with a recurrent *ETV6‐NTRK3* fusion gene and is not driven by PI3K pathway alterations [37]. Whether JP shows similar genetic alterations as or is clonally related to associated special types of breast carcinoma driven by other molecular mechanisms is uncertain.

Finally, we observed a greater exposure to an APOBEC signature in *in situ* and invasive carcinoma, compared with JP, which showed a dominant exposure to an aging signature. Our observations suggest that APOBEC mutational processes might underpin the evolution of JP to the associated DCIS and IDC‐NST components, in a way akin to the shifts from aging‐related signatures to APOBEC‐related mutagenesis in the progression from lobular carcinoma *in situ* to invasive lobular cancer [7] and from primary tumors to metastasis [38].

This study has several limitations, including its small sample size due to the rarity of JP and only one of three JPs analyzed was associated with *in situ* and invasive carcinoma. Although we provide a proof‐of‐principle that JP may constitute the substrate from which DCIS and invasive breast cancers may develop, further studies are required to ascertain the frequency of this phenomenon. Despite these limitations, here we confirm the presence of recurrent *PIK3CA* mutations in JP and provide evidence that JP and coexisting carcinoma are clonally related, further expanding our understanding of the relationship between JP and breast cancer.

## Author contributions statement

TMD, FP, EB, BW and JSR‐F conceived the study. TMD, EC and SAH provided samples. TMD, FP, MV, EC, SAH, YHW, EB and JSR‐F performed histopathological review. MV conducted the sample processing. ADCP, AG and LF performed the bioinformatics analysis. TMD, FP, ADCP, MV, AG, EMdS, BW and JSR‐F discussed and interpreted the results. TMD, FP and MV wrote the first draft. ADCP, AG, LF, EMdS, EC, LS, SC, HZ, SAH, YHW, EB, BW and JSR‐F read, edited, and approved the final manuscript.

## Data Availability

Whole‐exome sequencing data that support the findings of this study are available for visualization and download in cBioPortal for Cancer Genomics https://www.cbioportal.org/study/summary?id=brca_jup_msk_2020
